# Prostatic Cell-Specific Regulation of the Synthesis of MUC1-Associated Sialyl Lewis a

**DOI:** 10.1371/journal.pone.0057416

**Published:** 2013-02-22

**Authors:** Vishwanath B. Chachadi, Mohamed F. Ali, Pi-Wan Cheng

**Affiliations:** 1 Department of Research Service, Veterans Administration Nebraska-Western Iowa Health Care System, Omaha, Nebraska, United States of America; 2 Department of Biochemistry and Molecular Biology, College of Medicine, University of Nebraska Medical Center, Omaha, Nebraska, United States of America; 3 Eppley Institute for Research in Cancer and Allied Diseases, University of Nebraska Medical Center, Omaha, Nebraska, United States of America; The University of Kansas Medical Center, United States of America

## Abstract

Sialyl Lewis antigens are selectin ligands involved in leukocyte trafficking and cancer metastasis. Biosynthesis of these selectin ligands occurs by the sequential actions of several glycosyltransferases in the Golgi apparatus following synthesis of the protein backbone in the endoplasmic reticulum. In this study, we examine how the synthesis of sialyl Lewis a (sLe^a^) is regulated in prostatic cells and identify a mucin that carries this glycotope. We treat human prostatic cells including one normal and three cancerous cells with histone deacetylase inhibitors, valproic acid, tricostatin A (TSA), and suberoylanilide hydroxamic acid (SAHA), and then monitor the expression of sLe^a^. We have found that SAHA enhances the production of sLe^a^ in normal prostatic RWPE-1 cells but not prostatic cancer cells. Employing siRNA technology and co-immunoprecipitation, we show that the sLe^a^ is associated with MUC1, which is confirmed by confocal immunofluorescence microscopy and proximity ligation assay. The SAHA-induced production of sLe^a^ in RWPE-1 cells is resulted from upregulation of *B3GALT1* gene via enhancement of acetylated histone-3 and histone-4. Interestingly, PC3 and LNCaP C-81 cells do not produce detectable amounts of sLe^a^ despite expressing high levels of B3GALT1. However, the MUC1-associated sLe^a^ is generated in these cells after introduction of MUC1 cDNA. We conclude that the synthesis of sLe^a^ is controlled by not only peptide backbone of the glycoprotein but also glycoprotein-specific glycosyltransferases involved in the synthesis of sLe^a^. Further, the SAHA induction of this selectin ligand in normal prostatic cells may pose a potentially serious side effect of this drug recently approved by the US Food and Drug Administration.

## Introduction

**Figure 1:**
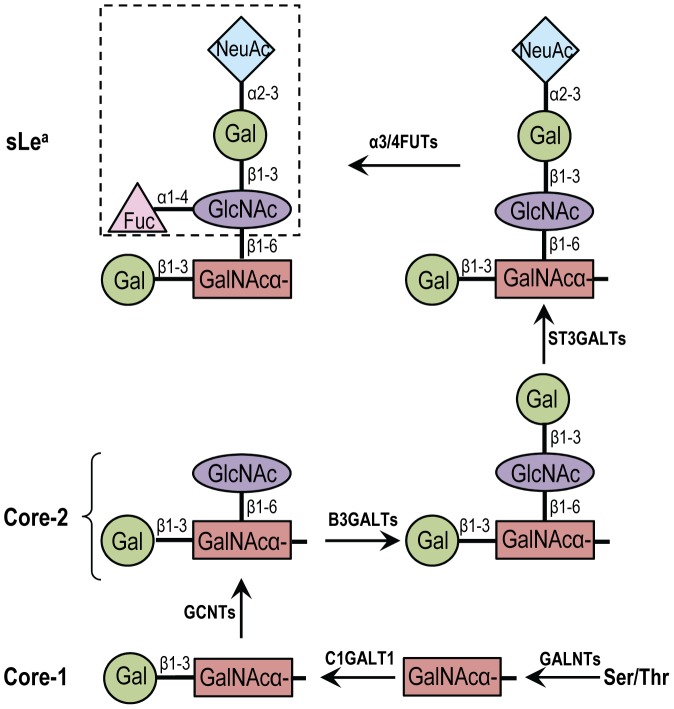
Biosynthetic pathway of nucin core 2-associated sialyl Lewis a (sLe^a^) antigen. Biosynthesis of mucin O-glycans is initiated by the addition of GalNAc to ser or thr of the peptide as catalyzed by polypeptide N-acetylgalactosaminyltransferases (GALNTs). This is followed by the addition of Gal in β1-3 linkage to GalNAc as catalyzed by C1GALT1 enzyme and produces core-1. Core 2 is generated by adding GlcNAc in β1-6 linkage to GalNAc by GCNTs. To synthesize sLe^a^, Gal is transferred to GlcNAc in β1-3 linkage to form type 1 chain as catalyzed by B3GALTs, followed by the addition of α2-3NeuAc to Gal as catalyzed by ST3GALTs and then α4-fucose (Fuc) to GlcNAc as catalyzed by α3/4FUTs.

Tumor metastasis is the primary cause of the mortality of cancer patients. The tumor invasion and metastasis properties acquired during cancer progression include increased invasion of surrounding tissues, escape from primary site, and establishment of tumors at distant sites. This process is driven by different families of adhesion molecules including integrins, members of the immunoglobulin superfamily, selectins and carbohydrate ligands, such as sialyl Lewis x (sLe^x^) and sialyl Lewis a (sLea) [1]. SLe^x^, NeuAcα2,3Galβ1,4(Fucα1,3)GlcNAcβ1→R, is a carbohydrate antigen expressed on neutrophils, monocytes, certain T lymphocytes, and advanced cancers, and plays a key role in leukocyte trafficking and cancer metastasis [2], [3]. This antigen has been used as a diagnosis and prognosis marker for cancer [4], [5], [6]. Similar to sLe^x^, sLe^a^, NeuAcα2,3Galβ1,3(Fucα1,4)GlcNAc→R, also known as CA 19.9, is widely expressed on tumors in the gastrointestinal tract and has been used as a marker for pancreatic and colon cancer [7], [8]. SLe^a^ is also a ligand for endothelial leukocyte adhesion molecule and is associated with metastasis [9], [10], [11] of human colon cancer [12], [13] and pancreatic adenocarcinoma [14]. Both sialyl Lewis antigens are found on various glycoproteins and mucins, including MUC1, which serve as selectin ligands to mediate leukocyte adhesion and hematogenous metastasis of cancer cells [15], [16], [17]. Biosynthesis of these ligands occurs by sequential actions of several glycosyltransferases with the final reactions completed by α2,3-sialyltransferases and then α1,3/1,4-fucosyltransferases [18]. Four α2,3-sialyltransferases (ST3GAL3-6) [19], [20] and four α3/4 fucosyltransferases (α3/4 FUT3-5 and -7) [21] can act on type I (Galβ1,3GlcNAcβ1-R) structure to generate sLea and on type II (Galβ1,4GlcNAcβ1-R) structure to produce sLex oligosaccharides despite different degrees of preference for their glycan substrates. The biosynthetic pathway for core 2-associated sLea is shown in figure 1. The major difference between sLea and sLex lies in the fucose linked to terminal GlcNAc through α1-4 linkage in sLea and α1-3 in sLex on type-1 and type-2 backbone structures, respectively. The type-1 backbone structure is synthesized by β1,3-galactosyltransferases (B3GALTs) while type-2 by β1,4-galactosyltransferases (B4GALTs).

The expression of glycosyltransferase genes can be regulated epigenetically. Epigenetics is a gene regulation mechanism without involvement of alterations in the DNA sequence [22]. The epigenetic changes such as DNA methylation and histone modification [23] have been shown to contribute to the development of malignancy-associated phenotypes such as growth, invasion, metastasis, or angiogenesis. DNA methylation is involved in the silencing of many glycosyltransferase genes, including heparin sulfate 3-O-sulfotransferase, *3-OST-2* [24], *ABO* [25], *B4GALNT2* [26], and *ST3GAL6* [19]. Histone modification also is involved in the silencing of many genes in colon cancer [27]. These results and our previous studies demonstrating the upregulation of sLe^x^ upon sodium butyrate or 5-Aza-2’-deoxycytidine (5-Aza-dC) treatment [19], [28] prompted us to examine the effect of other potential chemotherapeutic agents on the biosynthesis of sLe^a^ in prostatic cells. We treated immortalized normal and cancerous prostatic cells with various histone deacetylase inhibitors (HDACi) [29], including valproic acid, tricostatin A (TSA), and suberoylanilide hydroxamic acid (SAHA), and then monitored changes of sLe^a^ antigen. We found that normal prostatic cells were the only cells that were induced to produce sLe^a^ and SAHA was the only inhibitor that was capable of doing that. We showed that sLe^a^ was associated with MUC1, which was resulted from enhancement of *B3GALT1* gene expression through elevated levels of acetylated histone-3 (H3) and histone-4 (H4). We also found that PC3 and LNCaP C-81 cells were induced to produce MUC1-associated sLe^a^ after MUC1 cDNA was delivered to these cells. The results indicate that the ability of different prostatic cells to produce MUC1-associated sLe^a^ is controlled by the expression of either MUC1 or one of the glycosyltransferase genes involved in the synthesis of sLe^a^.

## Materials and Methods

### Materials

Keratinocyte serum-free medium (KSFM), bovine pituitary extract (BPE), epidermal growth factor (EGF) supplement, RPMI, penicillin, streptomycin, fetal bovine serum (FBS) and trypsin/EDTA were procured from Life Technologies Inc. (Grand Island, NY). Acrylamide and protein estimation kit were purchased from Bio-Rad (Hercules, CA). A mixture of pre-stained protein molecular weight standard marker was from Fermentas (Glen Burnie, MD). ECL reagent kit was obtained from Pierce Biotechnology (Rockford, IL). HDACi valproic acid and SAHA were from Cayman Chemical Company (Ann Arbar, MI) while TSA was obtained from Sigma (St. Louis, MO). Nuclear stain DAPI was from Life Technologies Inc. (Grand Island, NY). KM231 mouse monoclonal Abs to sLe^a^ was from EMD Millipore Corporation (Billerica, MA). Mouse monoclonal Abs to β-actin was from Sigma (St. Louis, MO). VU-4H5 mouse monoclonal Ab to MUC1 was obtained from Life Span Biosciences (Seattle, WA) and rabbit polyclonal Abs to MUC1 was from Abcam (Cambridge, MA). Abs to acetylated-H3, acetylated-H4 and for total histone proteins were obtained from Santa Cruz Biotechnology (Santa Cruz, CA). Abs against acetylated-H2A and H2B were purchased from Cell Signaling Technology (Beverly, MA). Horseradish peroxidase, DyLight® 488 (green) and DyLight® 595 (red) conjugated secondary antibodies (donkey anti-rabbit, donkey anti-mouse) were obtained from Jackson ImmunoResearch (West Grove, PA).

### Methods

#### Cell cultures and HDACi treatment

Immortalized normal human prostatic RWPE-1 cells were obtained from ATCC and grown in keratinocyte serum-free medium containing 0.05 mg/ml bovine pituitary extract and 5 ng/ml EGF. LNCaP C-81 cells derived from left supraclavicular lymph node metastasis were obtained from Dr. Ming-Fong Lin, prostatic cancer PC3 cells derived from bone metastasis (stage IV) and DU145 derived from brain metastasis were obtained from ATCC. These cells were cultured at 37 °C under 5% CO_2_ in RPMI-1640 medium containing 10% FBS and 100 U/ml penicillin and 100 µg streptomycin. For drug treatment, stock solutions of SAHA (1 mM) and valproic acid (1 M) were prepared fresh in sterilized PBS and TSA (50 µM) in DMSO within 1 h of the treatment. Final concentrations of HDACi were prepared by adding an appropriate amount of the stock solution directly to the culture medium. Drug and PBS (control) treatments of the cells were initiated at 50% confluence and then continued for 72 h.

#### Western blotting

Aliquots of the cell lysates from PBS or HDACi-treated cells were boiled for 5 min and then run on 4% SDS-PAGE (7.5×8.5 cm) under reducing conditions. After electrophoresis, the separated proteins were electro-transferred onto a PVDF membrane (Immobilon-P, 0.2 µ, Millipore, Bedford, MA), which was then blocked with tris-buffered saline containing 0.001% Tween 20 (TBST) and 5% (w/v) non-fat dried milk for 60 min. The membranes were then incubated with anti-sLe^a^ (KM231) (1∶500) or anti-MUC1 (VU-4H5) (1∶1,000) Abs overnight at 4 °C in same buffer. After five washings in same buffer, the membranes were treated for 1 h at room temperature (r.t.) with HRP-conjugated donkey anti-mouse IgG. The membranes were then washed five times (5 min each) with TBST and once with milli-Q water. Then, the blots were developed with ECL-sensitive film. For β-actin and acetylation analysis, proteins were separated on 10 and 15% SDS-PAGE, respectively and probed with respective primary and secondary Abs.

#### Immunoprecipitation (IP)

To isolate glycoproteins containing sLe^a^ ligand for characterization of conjugated glycans, cells were lysed in IP lysis buffer (Boston BioProducts, Worcester, MA) and precleared with protein G-agarose (Pierce). The precleared cell lysate (500 µg) was incubated with 5 µg of mouse anti-sLe^a^ (or anti-MUC1) mAb for 12 h at 4 °C. The immuno-complexes isolated with protein G-agarose for 1 h at 4 °C were analyzed for sLe^a^ by western blotting. The immunoprecipitates obtained with non-specific mouse IgG antibodies served as the negative control. Protein concentration was measured by Coomassie blue dye (Pierce).

#### Mixed glycosidases or Peptide-N-glycosidase (PNGase) F treatments

The anti-sLe^a^ immunoprecipitate was boiled (100 °C) for 10 min in 0.5% SDS and 1% β-mercaptoethanol and then treated with PNGase F (Sigma, USA) (5 U/mg protein) in 50 mM phosphate, pH 7.5 containing 1% NP-40 at 37 °C for 16 h. The immunoprecipitate was also treated with mixed glycosidases including neuraminidase (Glyko®; GK80040), β(1-4) galactosidase/β-N-acetylglucosaminidase (PRO-LINK Extender; GK80115), and O-glycanase (Glyko®; GK 80110) according to the manufacturer's instruction. The samples were analyzed by anti-sLe^a^ western blotting before and after treatment with glycosidases.

#### Transient transfection of RWPE-1 cells with *MUC1* or *B3GALT1* siRNA

Pool of three different siRNAs targeting *B3GALT1* (sc-105001; SantaCrutz Biotechnology), one custom synthesized siRNA targeting *MUC1* RNA and scrambled siRNA were purchased from Dharmacon (Waltman, MA) (See Table S1 for the nucleotide sequences of all three siRNAs). After treatment with 5 µM SAHA for 12 h, RWPE-1 cells at 50% confluence were transfected for 8 h in OPTI-MEM® reduced serum medium containing 100 nM siRNA and Lipofectamine RNAi MAX reagent (Life Technologies). The medium was then replaced with a fresh RPMI medium containing 10% FBS and 5 µM SAHA. After cultured for 52 h (total 72 h), the transfected cells were analyzed for *MUC1* and *B3GALT1* mRNA by quantitative PCR and sLe^a^ by western blotting as described above.

#### Confocal immunofluorescence microscopy

RWPE-1 cells were grown on cover slips in either presence or absence of 5 µM SAHA for 72 h. The cells were then fixed in 4% paraformaldehyde/PBS at r. t. for 30 min, washed thrice with PBS and blocked for 1 h in 3% normal donkey serum. After treated with mouse anti-sLe^a^ and rabbit anti-MUC1 Abs (1∶100) at 37 °C for 1 h, the cells were stained with DyLight 488 conjugated donkey anti-mouse Ab (green), and DyLight 594 conjugated donkey anti-rabbit Ab (red) (1∶200) and mounted in ProLong Gold antifade reagent with DAPI nuclear stain. Stained cells were examined on a Zeiss 510 Meta Confocal Laser Scanning Microscope and analyzed using Zeiss 510 software.

#### Flow cytometric analysis

Cell surface expression of sLe^a^ was quantitatively assessed by flow cytometry using anti-sLe^a^ antibodies [19]. Briefly, SAHA or PBS-treated RWPE-1 cells were dislodged, washed with PBS and washing buffer. The cells (0.2×10^6^) were blocked for non-specific staining in blocking buffer as described above and then incubated with anti-sLe^a^ Abs (10 µg/ml in washing buffer) on ice for 1 h. After thorough washing, secondary Ab staining was performed with DyLight 488 conjugated donkey anti-mouse IgG (1∶200) for 30 min on ice and excessive Abs were washed with washing buffer. Finally, cells were resuspended in 500 µl of PBS containing 0.5% paraformaldehyde and analyzed using a FACS Vantage (Becton Dickson, San Jose, CA) equipped with 488 nm argon laser and with Cell Quest-pro software. The unstained cells and cells incubated with secondary Ab alone served as negative and Ab controls, respectively.

#### In situ Proximity Ligation Assay (PLA)

*In situ* PLA was performed in RWPE-1 and PC3 cells treated with PBS or SAHA. These two cells were grown on cover slip in the presence or absence of 5 µM SAHA for 72 h. The cells were then fixed in 4% paraformaldehyde/PBS at r. t. for 30 min, washed thrice with PBS and blocked for 1 h in 3% BSA. After treatment with mouse anti-sLe^a^ and rabbit anti-MUC1 Abs (1∶100 in PBS with 3% BSA) at 37 °C for 1 h, cells were washed thrice (5 min each) with PBST. Oligonucleotide-conjugated anti-mouse minus and anti-rabbit plus PLA secondary probes were added at appropriate dilutions prepared in PBS with 3% BSA and the cells were incubated in a humidified chamber for 1 h at 37 °C. The PLA assay was performed using the Duolink PLA kit (Olink Bioscience cat# LNK-92101-KI01, Uppsala, Sweden) according to the manufacture's instruction. Briefly, connector oligonucleotides were hybridized and circularized by ligation for 30 min at 37 °C. After thorough washing, the cells were incubated with DNA polymerase for 100 min at 37 °C to produce rolling circle amplification products tagged with a red fluorescence probe. The nuclei were counterstained with DAPI, and the PLA signals were visualized at 60 x magnifications under Zeiss 510 Meta Confocal Laser Scanning Microscope equipped with DAPI/Texas Red filters and analyzed using Zeiss 510 software.

#### Quantitative real-time PCR analysis of glycosyltransferase and mucin genes

Quantitative real-time PCR analyses of various glycogenes were performed as described earlier [19]. In brief, all four cell lines cultured in T-25 flask were lysed with 1 ml TRI-REAGENT (Molecular Research Center, Inc, Cincinnati, OH) and mRNA was isolated according to the manufacturer's instruction. For cDNA synthesis, 2 µg RNA was used in a 20 µl reaction mixture using a Verso reverse transcriptase kit (Thermo scientific) as follows: 5 min at r.t., 60 min at 42 °C, and 2 min at 95 °C. Quantitative real-time PCR was performed in 10 µl reaction volume in a 96-well plate using 2 µl of diluted cDNA with SYBR® *Premix Ex Taq*™ (TAKARA BIO INC.) on a Mastercycler Epgradient realplex (Eppendorf AG, Hamburg, Germany). The PCR conditions included 1 cycle at 95 °C for 2 min followed by 45 cycles at 95 °C for 15 s, 60 °C for 15 s, and 72 °C for 45 s. The data were analyzed using Eppendorf realplex software, version 1.5 (Eppendorf). The amounts of various glycogene transcripts were normalized to the amount of *GAPDH* transcript in same cDNA sample. Relative fold differences in transcript expression of each target gene were determined using the comparative CT method: 2^-[ΔCt (SAHA)-ΔCt (PBS)]^ = 2^-ΔΔCt,^ where ΔC_t_ (in *PBS or SAHA-treated sample*) = C_t_ (in *PBS or SAHA-treated sample*)-C_t_ (*GAPDH in PBS or SAHA-treated sample*) as described by Tassone et al. [30]. The results were expressed as % of the target gene relative to that (100%) of *GAPDH* and plotted as mean fold changes ± standard error of the mean (SEM). Primer sequences used for expression analysis of all genes including *GAPDH* are summarized in Table S2.

#### Transient transfection of PC3 and LNCaP C-81 cells with MUC1 cDNA

Full length MUC1 cDNA containing N-terminal FLAG tag was prepared as previously described [31], [32]. Transient transfections of PC3 and LNCaP C-81 cells were carried out using the Lipofectamine 2000 (Life science technologies) following the manufacturer's protocol. Briefly, 1×106 cells/well in 6 well culture plates were incubated with 10 µg of MUC1F cDNA for 8 h in OPTI-MEM medium containing 15 µl of Lipofectamine 2000. After 8 h, the medium was replaced with complete PRMI containing 10% FBS and antibiotics. Cells were cultured for 72 h and harvested for western blot analysis.

#### Statistical analysis

All statistical analyses were carried out using the Student's t-test. SigmaPlot software (Systat Software, San Jose, CA) was used for all the graphing and statistical analyses. Data are expressed as mean ± SEM. *p* < 0.05 was considered statistically significant.

## Results

### SAHA treatment enhances the synthesis of sLe^a^ in RWPE-1 cells

**Figure 2:**
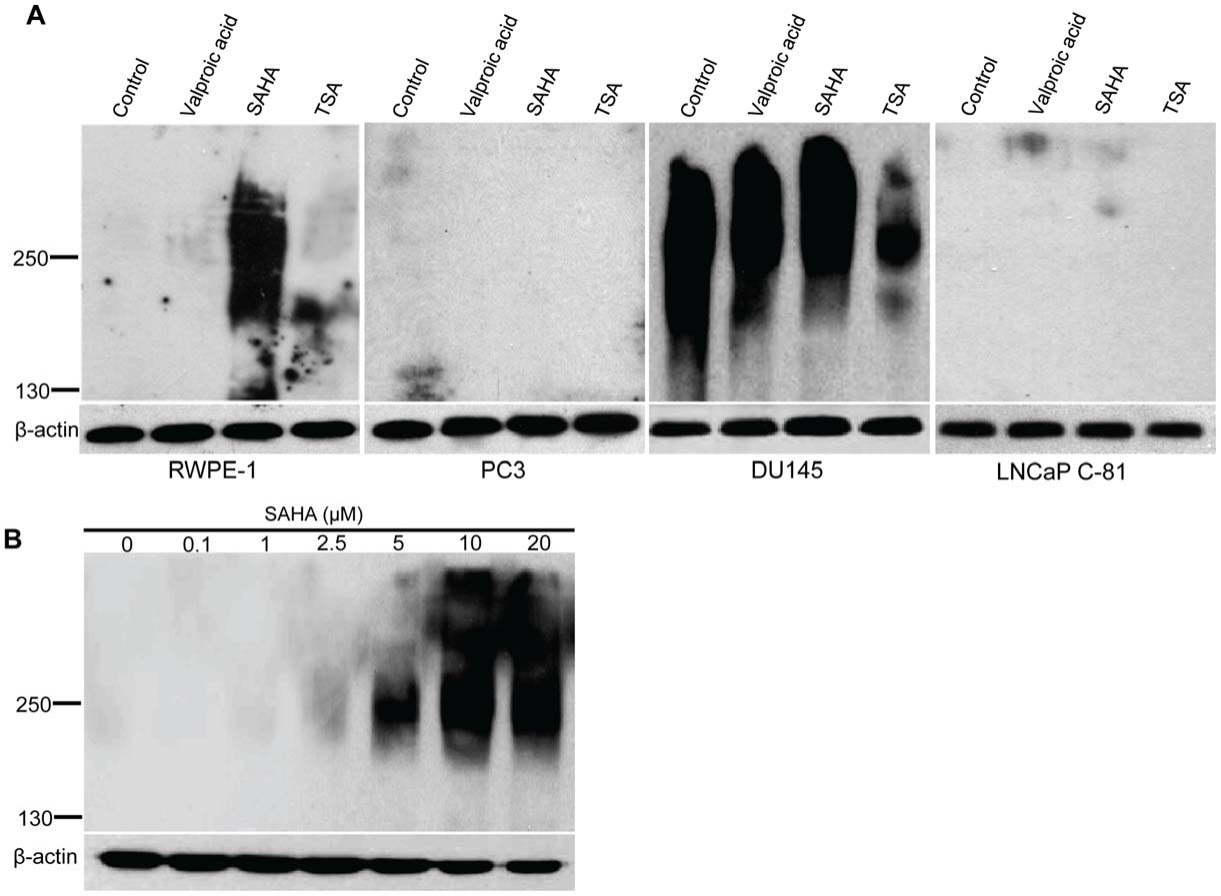
Effects of HDACi treatment on production of sLe^a^ in one normal and three cancerous prostatic cells. (A) RWPE-1, PC3, DU145, and LNCaP C-81 cells were treated with 1 mM valproic acid, 20 µM SAHA or 250 nM TSA for 72 h followed by western analysis of the cell lysates for sLe^a^ using KM231 Abs. SAHA was the only HDACi which induces marked production of sLe^a^ in RWPE-1 cells. (B) Dose-dependent (0-20 µM) effect of SAHA on the production of sLe^a^ in RWPE-1 cells. Minimum concentration of SAHA required to induce the production of sLe^a^ without causing cytotoxicity is 5 µM. β-actin was used as a loading control on 10% gels and probed with anti-β-actin Abs. Experiments were performed (3x) with similar results.

To study the effect of histone acetylation on the synthesis of sLe^a^, a ligand that mediates hematogenous metastasis of cancer cells, we treated three different prostatic cancer cell lines and one immortalized normal prostatic epithelial cell line with three different HDACi, valproic acid, SAHA, and TSA. We found that SAHA was the only one that enhanced the production of sLe^a^ in normal RWPE-1 cells (Fig. 2A). SLe^a^ was found to be associated primarily with a 250 kDa band. The SAHA effect was dose dependent (Fig. 2B). Because 5 µM SAHA was the lowest concentration that did not exhibit apparent cytotoxicity, it was used for subsequent experiments. It was noted that valproic acid showed no effect on all these cell lines tested. On the other hand, TSA inhibited the production of sLe^a^ in DU145 cells but did not show any effect on other cell lines. The cause for this inhibition is not known and will be a subject of future investigation.

### SLe^a^ is associated with O-glycans on MUC1 in RWPE-1 cells

**Figure 3:**
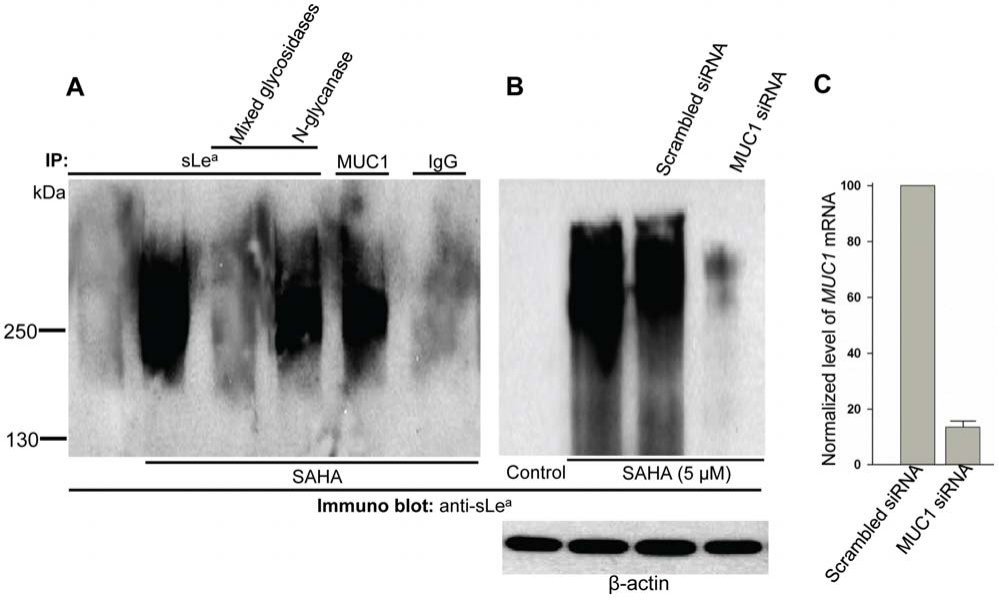
SLe^a^ induced by SAHA in RWPE-1 cells is associated with O-glycans of MUC1. Anti-sLe^a^ immunoprecipitates from the lysates of RWPE-1 cells treated with SAHA were digested with either mixed glycosidases (to remove sialic acid and O-glycans) or N-glycanase (which removes N-glycans) prior to western analysis for sLe^a^. (A) SLe^a^ band in mixed glycosidase-treated samples were completely abolished while sLe^a^ staining was retained after N-glycanase treatment. SLe^a^ band was also detected in anti-MUC1 immunoprecipitate (MUC1 IP), indicating that sLe^a^ is associated with MUC1. IgG lane served as a background control. (B) Anti-sLe^a^ western blot of the lysates of cells treated with PBS, SAHA, SAHA plus scrambled RNAi, or SAHA plus *MUC1* RNAi. (C) Relative expression level of *MUC1* gene was determined according to ΔCt method (see materials and methods) after normalization with *GAPDH* ( = 100%) and then expressed as % of *MUC1 mRNA* in the control ( = 100%) (n = 3).

To determine the nature of the SAHA-induced sLe^a^ in RWPE-1 cells, we treated the anti-sLe^a^ Ab pull-down with mixed glycosidases or N-glycanase. As shown in Fig. 3A, mixed glycosidases removed sLe^a^ epitope while N-glycanase did not, suggesting that the sLe^a^ is associated with O- and not N-glycans. Because the size of the sLe^a^ band (250 kDa) in the immunoprecipitate resembles that of MUC1, we predicted that the sLe^a^ might be associated with MUC1. This prediction was confirmed by the detection of sLe^a^ in anti-MUC1 Ab pull-down (Fig. 3A). Further, the sLe^a^ signal in SAHA-treated cells was greatly reduced after the *MUC1* mRNA had been knocked down by 86.6 ± 2.2% (Fig. 3B &C) confirming the above prediction. The results suggest that the sLe^a^ induced by SAHA treatment in RWPE-1 cells is associated with O-glycans on MUC1.

### The SAHA-induced sLe^a^ is on the surface of RWPE-1 cells

**Figure 4:**
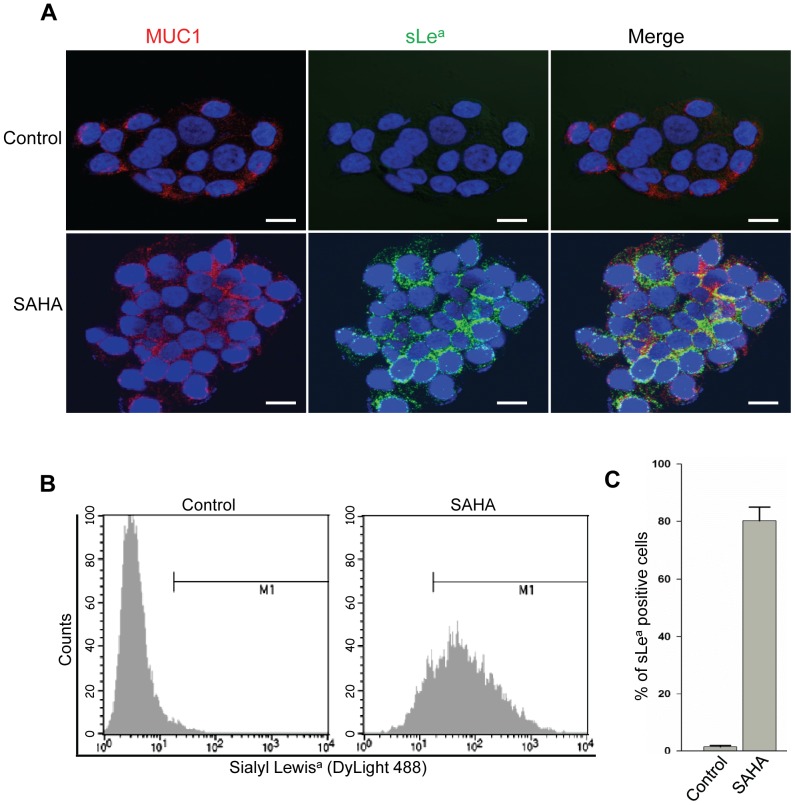
Confocal and flow cytometric analysis of RWPE-1 cells. (A) Confocal immunofluorescence images of MUC1 (red) and sLe^a^ (green) on RWPE-1 cells treated with PBS (control) or SAHA (5 µM) for 72 h. SLe^a^ induced by SAHA is colocalized with MUC1. (B) SLe^a^ mean fluorescence intensity in RWPE-1 cells treated with PBS or SAHA as measured by flow cytometry analysis. (p < 0.05) (C) % of sLe^a^-positive cells in PBS- or SAHA-treated cells. The data expressed as mean ± SEM were obtained from three independent experiments. (p < 0.05) Bar  =  20 µm.

By confocal immunofluorescence microscopy, we found that MUC1 co-localized with sLe^a^ induced by SAHA at the plasma membrane of RWPE-1 cells (Fig. 4A), validating the observation that MUC1 is the carrier of sLe^a^. These results were further confirmed by flow cytometric analysis performed on RWPE-1 cells with or without SAHA treatment. SAHA treatment increased the mean fluorescence intensity, an arbitrary unit for measuring the fluorescence intensity, from 3 in PBS-untreated cells to 132.2 ± 11 in SAHA-treated cells (Fig. 4B). The result is consistent with the acquisition of MUC1-associated sLe^a^ in SAHA-treated cells. Further, SAHA treatment increased the % of sLe^a^-positive cells from <1 to 82 ± 6% (Fig. 4C).

### *In situ* proximity ligation assay (PLA) of the colocalization of MUC1 and sLe^a^

**Figure 5:**
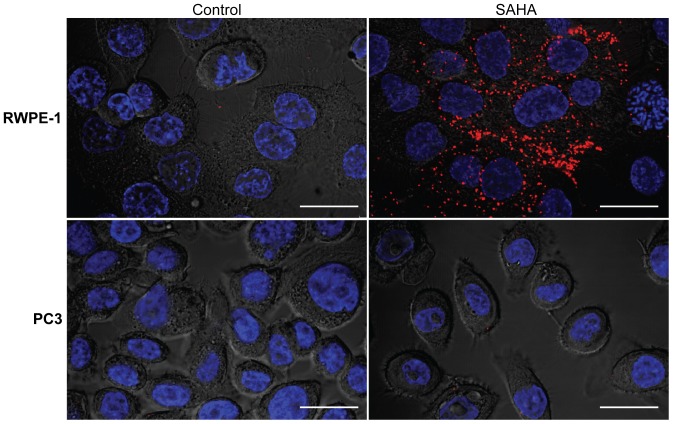
Proximity ligation assay of MUC1 and sLe^a^ in RWPE-1 and PC3 cells as analyzed by confocal microscopy. RWPE-1 and PC3 cells grown on cover slips were treated with PBS or SAHA and then fixed in paraformaldehyde. These cells were processed for PLA for MUC1 and sLe^a^ using an Olink Bioscience PLA kit. Briefly, these cells were treated with mouse anti-sLe^a^ and rabbit anti-MUC1 antibodies, and then with oligonucleotide-conjugated anti-mouse minus and anti-rabbit plus proximity ligation assay secondary probes. The oligonucleotides conjugated with the plus and minus antibodies were used to generate circular DNA, which was then amplified and tagged with a red fluorescence dye. Following DAPI staining of the nuclei, the cells were examined by confocal fluorescence microscopy. The expression as well as association of sLe^a^ with MUC1, significantly increased only in SAHA treated RWPE-1 cells as indicated by red foci all over the cells. Bar  =  20 µm.

The *in situ* PLA was performed to show that the newly synthesized sLe^a^ induced by SAHA is associated with MUC1. As shown in Fig. 5, after SAHA treatment, RWPE-1 cells produced sLe^a^ on MUC1 as shown by positive red dots in these cells while no red dots were detected in PBS-treated control cells. Similarly, as expected, both PBS-treated and SAHA-treated PC3 cells did not show any red dots. The SAHA-treated cells exposed to PLA probe, mouse anti-sLe^a^ Ab, or rabbit anti-MUC1 Ab, which served as negative controls, did not show any red dots, indicating clean background signals (Fig. S1).

### Enhanced *B3GALT1* gene expression is responsible for the synthesis of sLe^a^ in RWPE-1 cells treated with SAHA

**Figure 6:**
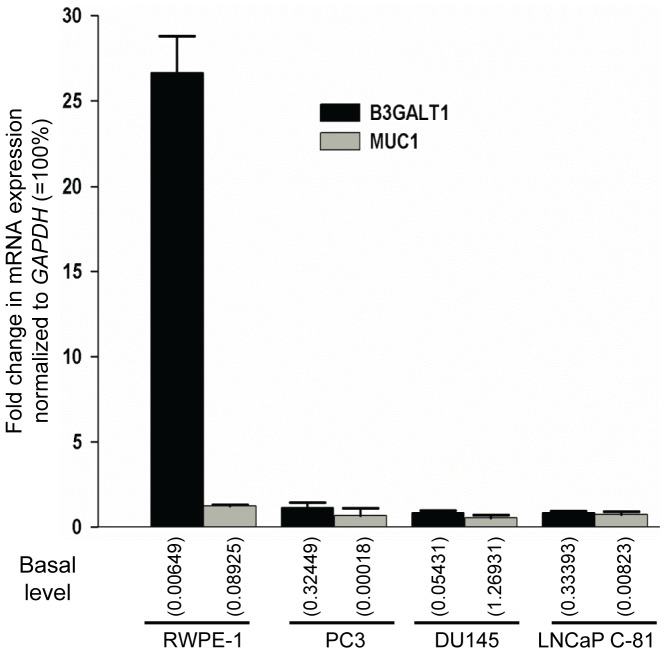
SAHA treatment stimulates the expression of *B3GALT1* gene in RWPE-1 cells. Quantitative real-time PCR analysis was carried out on RWPE-1, PC3, DU145 and LNCaP C-81 cells treated with PBS or 5 µM SAHA for 72 h. Relative expression levels of *B3GALT1* and *MUC1* genes were sorted according to ΔCt (see Materials and Methods) method, normalized with *GAPDH* in same cell preparation and expressed as fold changes ± SEM determined by calculating the ratio of the expression level of each gene in SAHA-treated vs. that in PBS-treated control cells. Relative amount of each gene versus that of *GAPDH* (100%) in PBS-treated control cells was given in the parenthesis (n = 3). The numbers shown in parenthesis represent % basal expression level relative to GAPDH ( = 100%).

To examine how SAHA treatment enhanced the synthesis of MUC1-associated sLe^a^ in RWPE-1 cells but did not affect the expression of sLe^a^ in the prostatic cancer cells, we performed real time PCR analyses to determine the expression levels of *MUC1* gene and glycosyltransferase genes involved in the synthesis of sLe^a^ in these cells treated with PBS or SAHA. As shown in Fig. S2 & S3, the glycogenes we analyzed include *C1GALT1*, three *GCNTs*, three *ST3GALs*, six *FUTs*, four *B3GALTs*, fourteen *GALNTs* and four membrane-bound *MUCs*. We found that the expression level of the *B3GALT1* gene was increased 26.6-fold from 0.006% to 0.173% of the expression level of *GAPDH* gene (Fig. 6), suggesting that this is the key enzyme involved in the synthesis of sLe^a^ induced by SAHA in RWPE-1 cells. The basal expression level (0.09% of *GAPDH*) of *MUC1* gene was much higher than the expression levels (< 0.0007% of *GAPDH*) of the other three MUC genes (*MUC4, MUC16 and MUC17*)(Fig. S2). SAHA treatment did not show an appreciable effect on the expression of all these *MUC* genes. Although *MUC1* gene was not enhanced in the SAHA-treated RWPE-1 cells, the basal expression level of this gene was sufficiently high (0.09% of *GAPDH*) that it was not considered a limiting factor for the synthesis of MUC1-associated sLe^a^ in these cells. However, in the prostatic cancer cells, the basal expression level of the *B3GALT1* gene was from moderate (0.05% of *GAPDH*) in DU145 cells to very high in the other two prostatic cancer cells, i.e. 0.324% of *GAPDH* in PC3 cells and 0.334% of *GAPDH* in LNCaP C-81 cells (Fig. 6), suggesting that this enzyme is not a limiting factor for the synthesis of sLe^a^ in these cells. Further, the *MUC1* gene expression level was very high in DU145 cells, i.e. 1.27% of *GAPDH*, and very low in PC3 cells, i.e. 0.0002% of *GAPDH*, and LNCaP C-81 cells, i.e. 0.008%, suggesting that the low expression level of *MUC1* gene is a limiting factor for the synthesis of sLe^a^ in these two prostatic cancer cells.

### *B3GALT1* gene participates in the synthesis of sLe^a^ on MUC1

**Figure 7:**
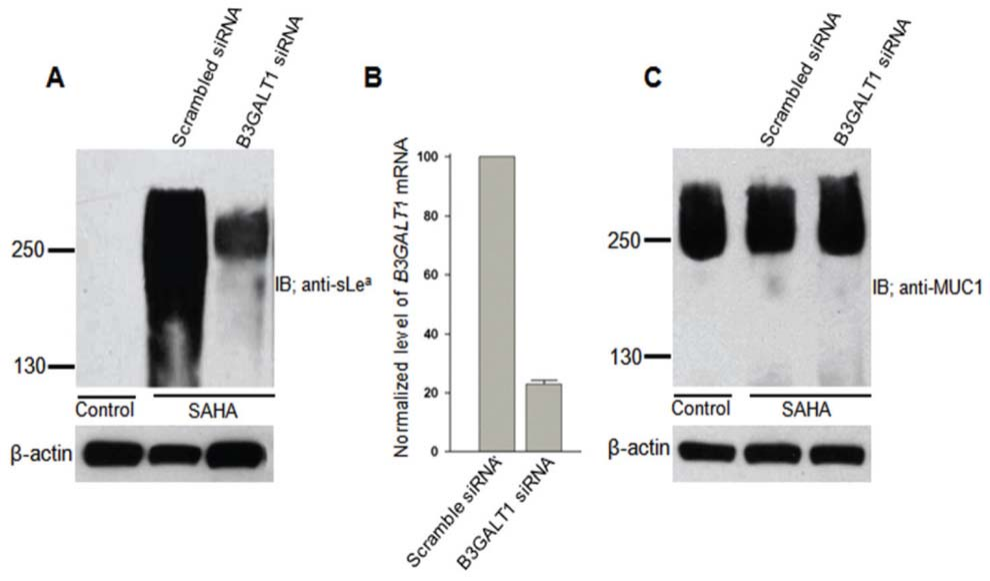
Knockdown of *B3GALT1* mRNA reduces sLe^a^ in SAHA-treated RWPE-1 cells. (A) Anti-sLe^a^ western blot of the lysates of control cells, SAHA plus scrambled siRNA, or SAHA plus *B3GALT1* siRNA. (B) Knockdown of *B3GALT1* gene in RWPE-1 cells was confirmed by 77% reduction of *B3GALT1 mRNA* measured by quantitative real-time PCR analysis. Relative expression level of *B3GALT1* gene was determined according to ΔCt method (see materials and methods) after normalization with *GAPDH* ( = 100%) and then expressed as % of *B3GALT1* mRNA relative to same in the control ( = 100%) (n = 3). (C) Anti-MUC1 western blot of the lysates of cells treated with SAHA, SAHA plus scrambled siRNA, or SAHA plus *B3GALT1* siRNA. Knockdown of 77% of *B3GALT1* mRNA reduces sLe^a^ in SAHA-treated RWPE-1 cells without affecting MUC1 glycoprotein. Experiments were performed (3x) with similar results.

To prove that B3GALT1 is involved in the synthesis of sLe^a^ on MUC1, we performed a *B3GALT1* mRNA knockdown experiment. As shown in Fig. 7A & B, knockdown of the *B3GALT1* mRNA by 77% greatly reduced the sLe^a^ signal. The reduction of sLe^a^ signal in *B3GALT1* gene knocked down cells did not affect the amount of MUC1 (Fig. 7C), indicating that the reduction of sLe^a^ signal in these cells is due to reduction of the synthesis of this epitope and not reduction of MUC1 protein.

### Transfection of PC3 and LNCaP C-81 cells with MUC1 cDNA results in the production of sLe^a^

**Figure 8:**
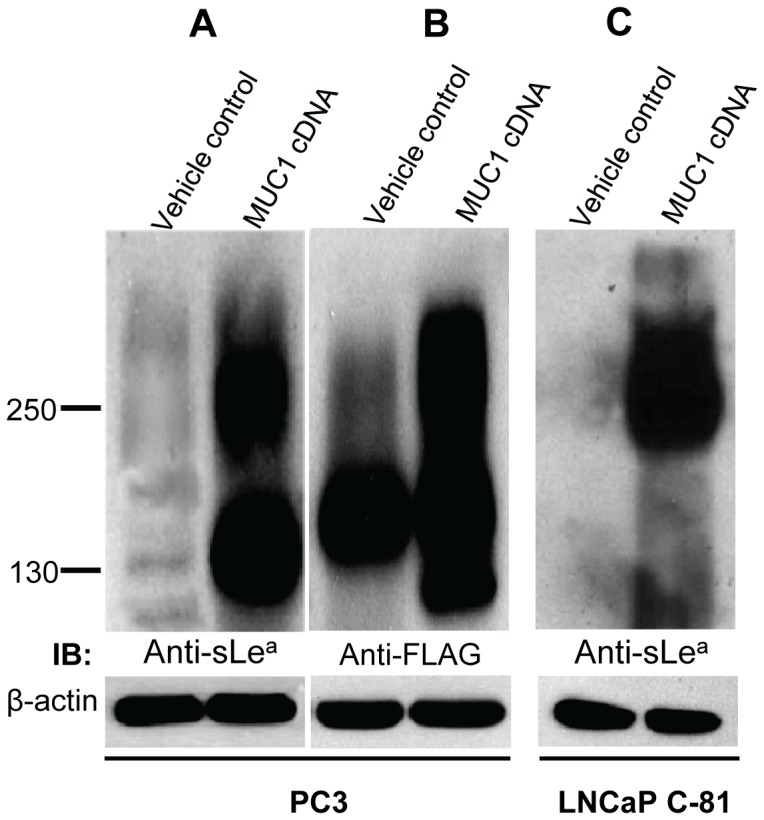
Transfection of PC3 and LNCaP C-81 cells with MUC1 cDNA results in production of sLe^a^. Full length MUC1 cDNA composed of tandem repeats and N-terminal FLAG epitope was used to transfect PC3 and LNCaP C-81 cells. After 72 h, cell lysates from both vehicle control and MUC1 cDNA-transfected cells were analyzed by sLe^a^ (KM231) western blotting. The MUC1 band corresponding to 250 kDa appeared after MUC1 cDNA transfection in both cell lines. The 130 kDa band in frame A probably represents truncated MUC1 in MUC1 cDNA-transfected cells. Antibodies did not detect any sLe^a^ band in vehicle-treated cells. For equal protein loading, β-actin was used and run on 12% SDS-PAGE.

To prove that the failure of PC3 and LNCaP C-81 cells to produce MUC1-associated sLe^a^ is the result of the lack of MUC1 expression, we monitored MUC1 as well as sLe^a^ generated in these cells after transfection of both cells with a plasmid containing MUC1 cDNA expressing a FLAG tag as described previously [31]. As shown in Fig. 8, sLe^a^ epitope was detected in a 250 kDa band and a 130 kDa band in MUC1 cDNA transfected PC3 cells (Fig. 8A & B). This 130 kDa band likely represents a truncated MUC1. Similar expression of sLe^a^ was also observed in LNCaP C-81 cells after introduction of MUC1 cDNA (Fig. 8C). The results indicate that the low level of *MUC1* gene expression is the limiting factor for the synthesis of MUC1-associated sLe^a^ in PC3 and LNCaP C-81 cells under basal conditions.

### H3 and H4 histone deacetylation is involved in the regulation of the expression of sLe^a^ in RWPE-1 cells

**Figure 9:**
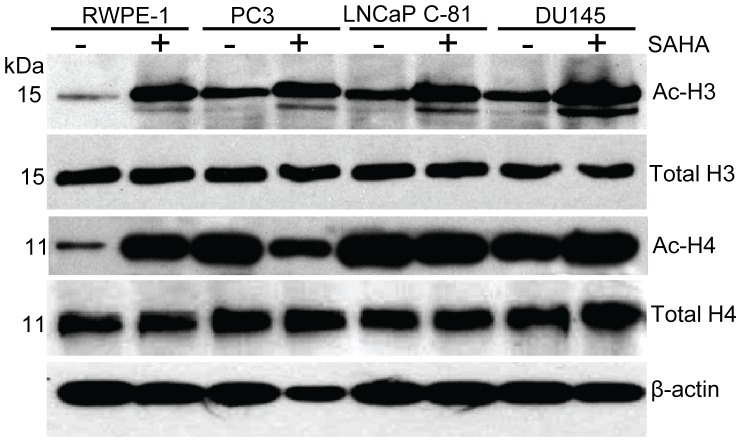
SAHA treatment of RWPE-1 cells increases acetylated H3 and H4 histones. Cell lysates were prepared from RWPE-1, PC3, LNCaP C-81 and DU145 cells treated with PBS or 5 µM SAHA for 72 h. Proteins (100 µg) were separated on 15% SDS-PAGE and blotted onto a PVDF membrane. The acetylated histone-3 and histone-4 proteins were detected with H3 and H4-specific antibodies along with total histone protein antibodies. The β-actin from same samples was used as a protein loading control.

To assess the role of histone acetylation in the B3GALT1-mediated sLe^a^ synthesis, we compared the levels of acetylated histones in these prostatic cells treated with PBS or SAHA. As shown in Fig. 9, all these prostatic cells showed up-regulation of the levels of acetylated H3 histone although the basal level of acetylated H3 histone in RWPE-1 cells was lower than those of the other three prostatic cells. Similarly, the basal level of acetylated H4 histone in RWPE-1 cells was lower than those in other prostatic cells. But, SAHA treatment greatly up-regulated the levels of acetylated H4 histone in RWPE-1 cells but not the other prostatic cells. However, we did not observe any increase in acetylation of H2A and H2B in RWPE-1 and DU145 cells but a small increase in the acetylation of H2A and H2B was observed in LNCaP C-81 and PC3 cells, respectively (Fig. S4). The results suggest that SAHA treatment inhibits histone deacetylase in RWPE-1 cells, which results in elevation of acetylated H3 and H4 histones, activation of the *B3GALT1* gene, and then induction of sLe^a^ decorated on MUC1.

## Discussion

SLea and sLex are carbohydrate antigens serving as ligands for selectins to mediate adhesion of cancer cells to the endothelium, thereby facilitating hematogenous metastasis [33], [34]. Synthesis of these antigens requires a tight coordination of several glycosyltransferases. Some of these glycosyltransferase genes are subject to epigenetic regulation. For example, we previously showed that the synthesis of MUC1-associated sLex in HCT15 colon cancer cells was controlled by methylation of the ST3GAL6 promoter [19]. The expression of many other glycosyltransferase genes is also under the control of DNA methylation [26], [35], [36], [37], [38], [39]. Apart from glycosyltransferases, many other glycogenes, including enzymes involved in the biosynthesis of sugar nucleotides [40], [41], galectins [42], and some transporters [27], also are regulated by epigenetics. Although these studies demonstrate epigenetic regulation of glycogenes and their phenotypes in cancer cells, no attempts have been made to investigate normal counterparts. Our study provides evidence that the sLe^a^ induced by SAHA decorates MUC1 as demonstrated by proximity ligation, confocal microscopy, and flow cytometry assays. Current study also shows that the synthesis of MUC1-associated sLea in human normal prostatic RWPE-1 cells is regulated by the B3GALT1 gene expression via histone acetylation.

Biosynthesis of a given mucin-associated glycan requires not only the mucin protein backbone but also all the glycosyltransferases needed for the synthesis of this glycan. Missing either the mucin protein backbone or any one of these glycosyltransferases would make the cells incapable of producing either this mucin at all or this mucin without the specific glycan, respectively. In our study, we show that one normal prostatic epithelial cell line can produce MUC1 protein and all the glycosyltransferases needed for the synthesis of sLea except B3GALT1. Activation of B3GALT1 gene with the HDACi SAHA makes it possible for the normal prostatic epithelial cells to generate MUC1 decorated with sLea. On the other hand, PC3 and LNCap C-81 cells which do not express sufficient amounts of MUC1 to allow the production of detectable amount of MUC1 to be decorated with sLea despite the presence of all the enzymes needed for the synthesis of this glycan. Providing MUC1 cDNA to PC3 and LNCaP C-81 cells makes it possible for these cells to produce sufficient amounts of MUC1 to be decorated with sLea. On the other hand, DU145 cells produce high levels of sLea irrespective of SAHA treatment as expected from high basal levels of both MUC1 and B3GALT1 enzyme. These results indicate that each cell type exhibits its own unique expression pattern of mucin and glycosyltransferase genes. Understanding the expression pattern of these genes in a given cell type would afford an opportunity for modulating the synthesis of a specific glycan decorated on a specific mucin in each cell type.

It is of interest to note that without SAHA treatment, RWPE-1 cells could not generate MUC1-associated sLe^a^ despite having a very high expression level of the *B3GALT5* gene, i.e. 1.04% of *GAPDH*. This enzyme has been shown to be involved in the synthesis of sLe^a^ [43], [44] on CD43 [45] and carcinoembryonic antigen [46], but does not work on MUC1. The results support the idea that each glycosyltransferase has its own set of glycoprotein substrates because each Golgi glycosyltransferase is localized to a specific Golgi compartment according to the glycosylation step in which it participates and also, each glycoprotein has its unique transport path as it is moved from cis to trans-Golgi. Therefore, only the glycoproteins that come in contact with specific glycosyltransferases as they travel through the Golgi apparatus can be decorated with the carbohydrate structures specified by these glycosyltransferases. The important implication of this glycosylation mechanism is that one glycosyltransferase isozyme may not substitute another isozyme even though they show similar substrate specificity when measured *in vitro*.

A recent report showed that the expression of MUC1 gene in prostatic cancer cells is inversely regulated by the androgen receptor (AR) in a dose-dependent manner [47]. A similar study also demonstrated that high levels of AR inhibited the production of MUC1 and AR-negative prostatic cancer cells expressed high level of MUC1 [48]. This study showed that AR suppressed the expression of MUC1 gene by inhibiting MUC1 promoter and reducing the translation of MUC1 messages by miR-125b induced by AR. However, this study focused only on cancer cells and not non-cancerous prostatic RWPE-1 cells. In our study, we found that RWPE-1 cells, an AR positive cell line, expressed MUC1, a phenomenon contrary to the proposed mode of action of AR in prostatic cancer cells. In addition, the report of Singh et al. [49] also confirms that RWPE-1 cells express MUC1. Our results indicate that the proposed mechanisms for the AR inhibition of MUC1 [48] do not apply to normal prostatic RWPE-1 cells. The mechanism of the failure of AR to inhibit MUC1 production in normal prostatic cells remains to be elucidated.

Histone acetylation and DNA methylation are two well established epigenetic modifications that are targeted by drugs known as HDACi and DNA methyltransferase inhibitors [23], respectively. Numerous HDACi have been developed and many of them have been tested in preclinical and early clinical studies [50]. Several structural classes of HDACi have been identified including short chain fatty acids (butyrate and valproic acid), hydroxamic acids (TSA and SAHA), cyclic tetrapeptides (trapoxin A and apicidin) and benzamides (MS-27-275). Both hydrophobic backbone and carboxyl group of valproic acid play key roles in the inhibition of HDAC [51]. On the other hand, the hydroxamate inhibitors have three common structural characteristics: a zinc binding moiety, an opposite capping group, and a straight chain alkyl, vinyl or aryl linker connecting the two. These functional groups have been shown to interact with the conserved region of the active site of various HDACs to inhibit the activity [52], [53]. SAHA, one of the hydroxamate inhibitors, is non-selective [54] and believed to be a potent inhibitor of class I and class II HDAC enzymes [55]. In our study, we observed upregulation of sLe^a^ only in SAHA-treated cells whereas other drugs (valproic acid and TSA) were ineffective. Given that valproic acid is a specific inhibitor of class I HDACs [56], and SAHA and TSA inhibit both class I and II HDACs [55], the SAHA effect probably occurs through inhibition of class II HDACs. Further, TSA induces histone acetylation transiently [57], [58] while SAHA-induced acetylation of histone-3 is long-lasting [58], which could explain the SAHA-induced sLe^a^ in RWPE-1 cells is likely the result of sustained inhibition of class II HDACs. In addition, it should be mentioned that acetylation of H3 and H4 and not that of H2A and H2B histones is involved in the activation of *B3GALT1* gene in these cells.

SAHA has been shown to inhibit the growth of LNCaP and PC3 cells and also shrink tumors and suppress their growth in mice transplanted with CWR22 human prostate tumor cells [59]. But, its effect on normal prostatic cells has never been reported until now. Our results showing that SAHA can induce the production of a metastasis-promoting selectin ligand sLe^a^ in normal prostatic cells is a cause of concern given that this drug has recently been approved by the US Food and Drug Administration for treating cutaneous T-cell lymphoma [60]. Should this observation be reproduced in additional normal prostatic cells, this side effect needs to be carefully monitored when this drug is used for cancer treatment.

## Supporting Information

Figure S1 **Negative control experiments for proximity ligation assay of MUC1 and sLe^a^ in SAHA-treated RWPE-1 cells.** RWPE-1 cells treated with 5 µM SAHA for 72 h were exposed to (A) PLA probe only, (B) mouse anti-sLe^a^ Ab plus rabbit IgG, or (C) rabbit anti-MUC1 Ab plus mouse IgG and then examined by confocal fluorescence microscopy after PLA assay described in the materials and methods. Bar = 20 µm
(DOCX)

Figure S2 **Quantitative real-time PCR analysis of membrane-bound mucin and glycosyltransferase genes.** Quantitative real-time PCR analysis was carried out on RWPE-1, PC3, DU1-45 and LNCaP C-81 cells treated with PBS or 5 µM SAHA for 72 h. Relative expression levels of mRNA of different genes were sorted according to ΔCt (see Materials and Methods) method, normalized with *GAPDH* in same cell preparation and expressed as fold changes ± SEM and then determined by calculating the ratio of the expression level of each gene in SAHA treated vs. that in PBS-treated control cells. Relative amount of each gene versus that of *GAPDH* (100%) in PBS-treated control cells was given in the parenthesis (n = 3).
(DOCX)

Figure S3 **Quantitative real-time PCR analysis of mucin Polypeptide N-acetylgalactosaminyltransferase (GALNT) mRNAs.** Fourteen different *GALNTs* were analyzed by quantitative real-time PCR in RWPE-1 cells treated with PBS or 5 µM SAHA for 72 h. Relative expression level of each GALNT was calculated and plotted as described above.
(DOCX)

Figure S4 **Effect of SAHA treatment on levels of acetylated H2A and H2B in RWPE-1 and prostatic cancer cells.** Lysates were prepared from RWPE-1, PC3, LNCaP C-81 and DU145 cells treated with PBS or 5 µM SAHA for 72 h. Proteins (100 µg) were separated on 15% SDS-PAGE and blotted onto a PVDF membrane. The acetylated H2A and H2B proteins were detected with respective antibodies. The β-actin from same samples was used as a protein loading control.
(DOCX)

Table S1 **Short interfering RNA (siRNA) sequences.**
(DOCX)

Table S2 **Oligonucleotide primers used for quantitative real-time PCR analysis.**
(DOCX)
