# Supplementary figures and images for: Prostatic Cell-Specific Regulation of the Synthesis of MUC1-Associated Sialyl Lewis a
Source: PLoS One. 2013 Feb 22;8(2):e57416. doi: 10.1371/journal.pone.0057416 (PMC3579856; doi:10.1371/journal.pone.0057416)

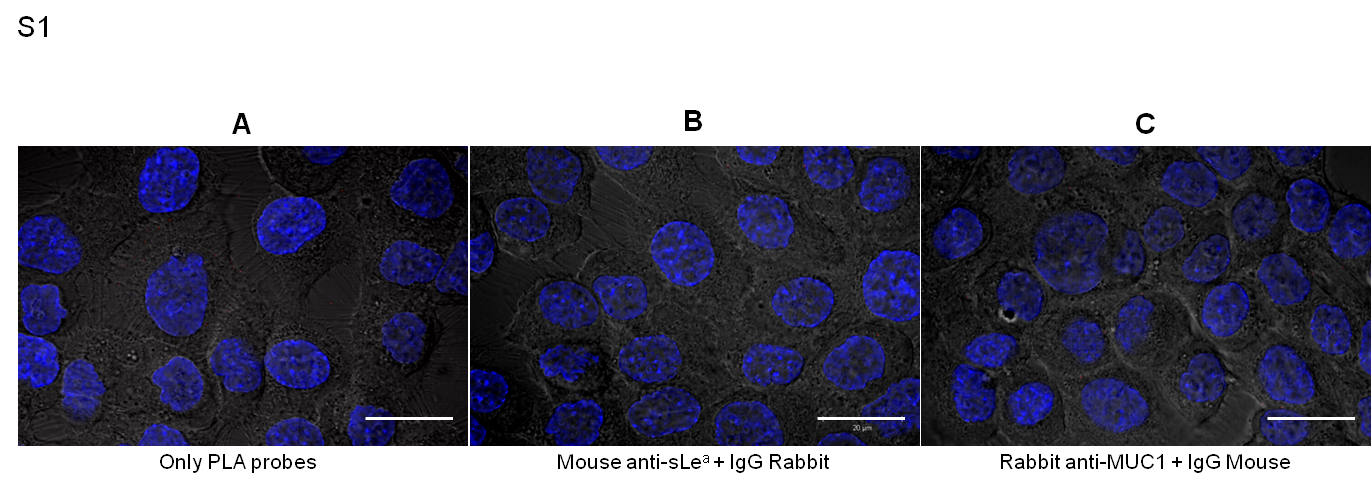

Supplement: Figure S1 — Negative control experiments for proximity ligation assay of MUC1 and sLea in SAHA-treated RWPE-1 cells. RWPE-1 cells treated with 5 µM SAHA for 72 h were exposed to (A) PLA probe only, (B) mouse anti-sLea Ab plus rabbit IgG, or (C) rabbit anti-MUC1 Ab plus mouse IgG and then examined by confocal fluorescence microscopy after PLA assay described in the materials and methods. Bar = 20 µm (DOCX) [file pone.0057416.s001.docx]

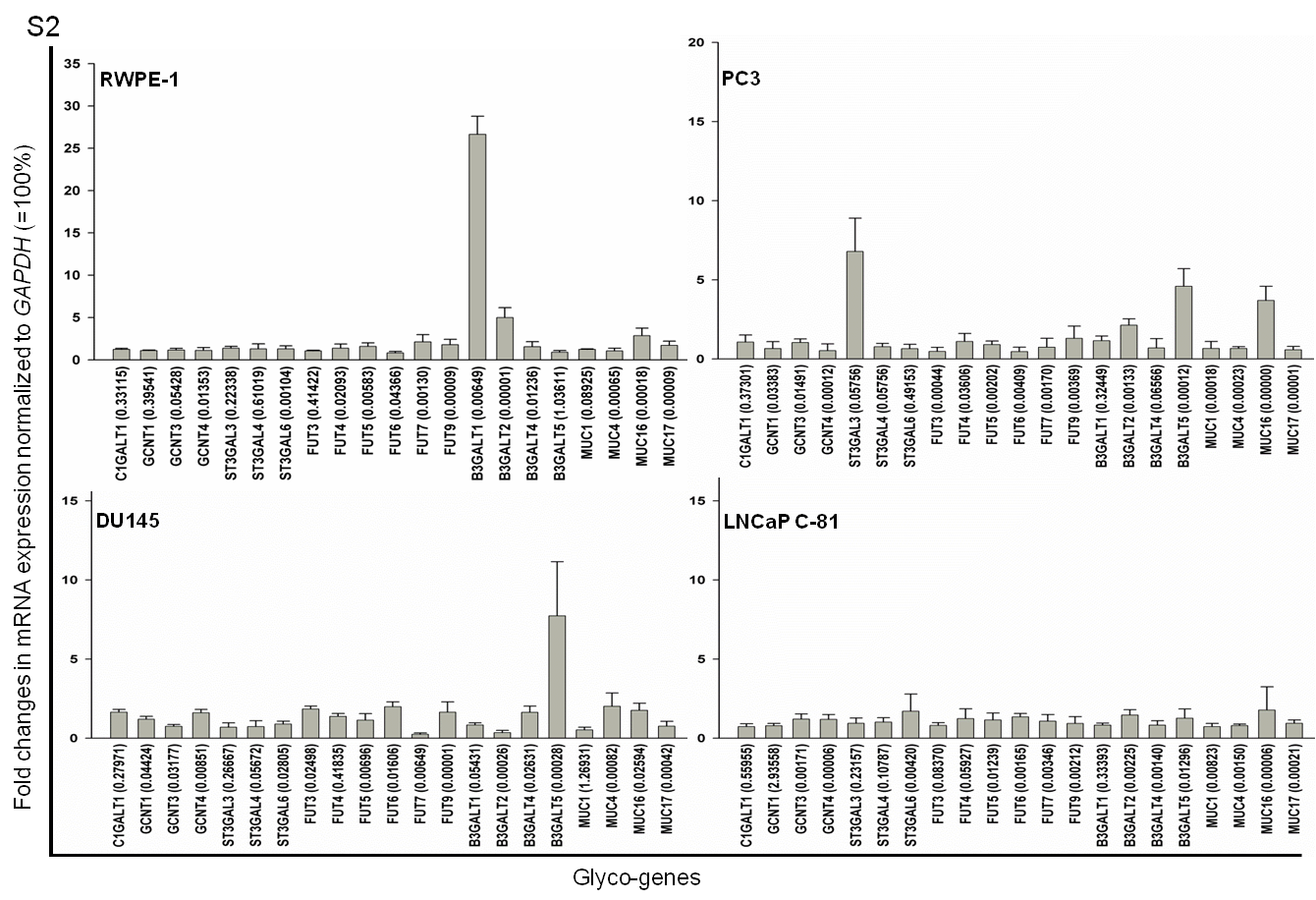

Supplement: Figure S2 — Quantitative real-time PCR analysis of membrane-bound mucin and glycosyltransferase genes. Quantitative real-time PCR analysis was carried out on RWPE-1, PC3, DU1-45 and LNCaP C-81 cells treated with PBS or 5 µM SAHA for 72 h. Relative expression levels of mRNA of different genes were sorted according to ΔCt (see Materials and Methods) method, normalized with GAPDH in same cell preparation and expressed as fold changes ± SEM and then determined by calculating the ratio of the expression level of each gene in SAHA treated vs. that in PBS-treated control cells. Relative amount of each gene versus that of GAPDH (100%) in PBS-treated control cells was given in the parenthesis (n = 3). (DOCX) [file pone.0057416.s002.docx]

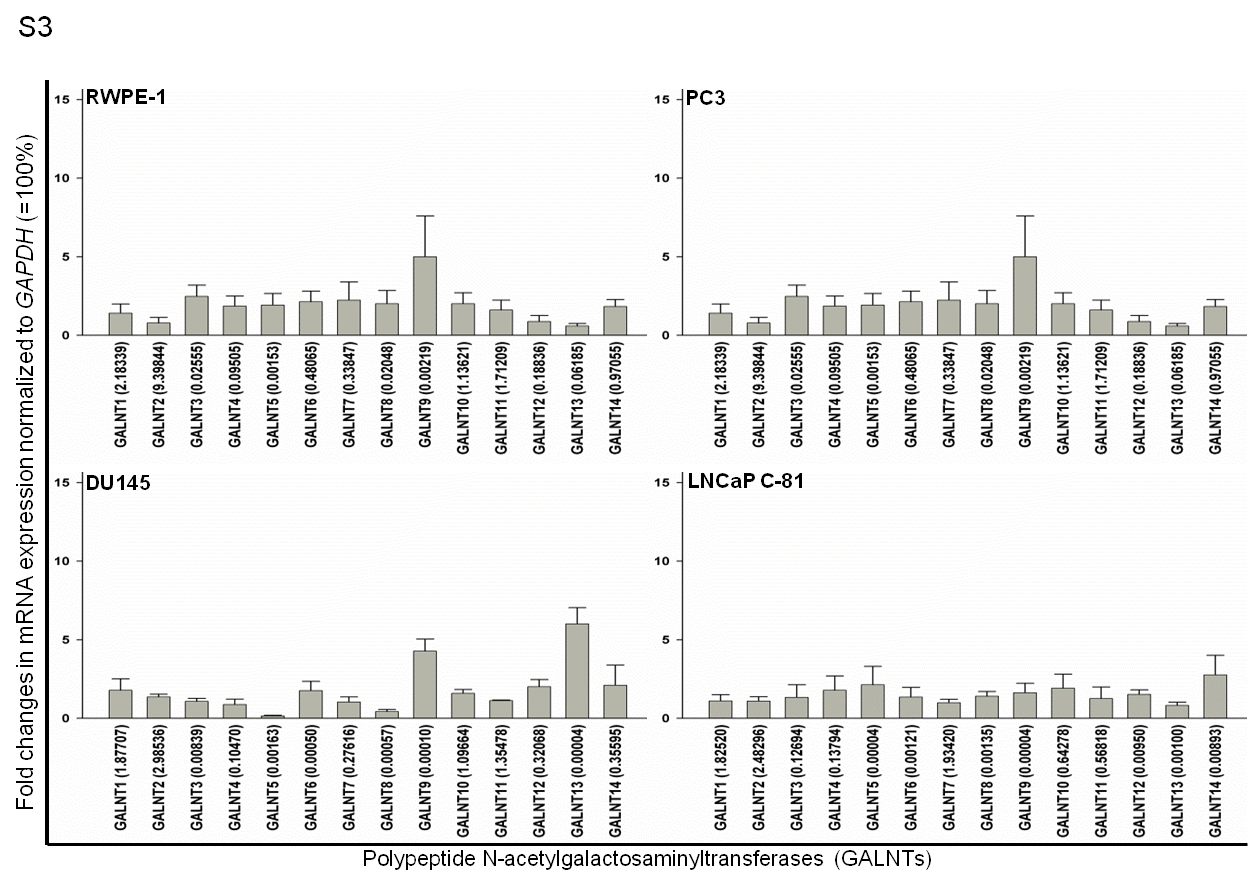

Supplement: Figure S3 — Quantitative real-time PCR analysis of mucin Polypeptide N-acetylgalactosaminyltransferase (GALNT) mRNAs. Fourteen different GALNTs were analyzed by quantitative real-time PCR in RWPE-1 cells treated with PBS or 5 µM SAHA for 72 h. Relative expression level of each GALNT was calculated and plotted as described above. (DOCX) [file pone.0057416.s003.docx]

**
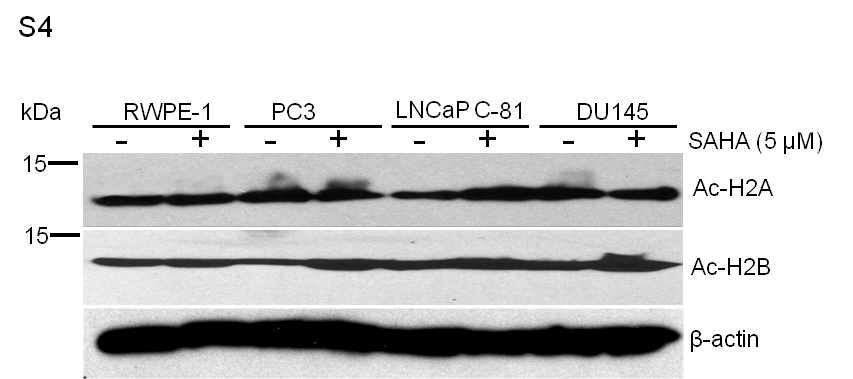
**

Supplement: Figure S4 — Effect of SAHA treatment on levels of acetylated H2A and H2B in RWPE-1 and prostatic cancer cells. Lysates were prepared from RWPE-1, PC3, LNCaP C-81 and DU145 cells treated with PBS or 5 µM SAHA for 72 h. Proteins (100 µg) were separated on 15% SDS-PAGE and blotted onto a PVDF membrane. The acetylated H2A and H2B proteins were detected with respective antibodies. The β-actin from same samples was used as a protein loading control. (DOCX) [file pone.0057416.s004.docx]
