# Supplementary material for: Prostatic Cell-Specific Regulation of the Synthesis of MUC1-Associated Sialyl Lewis a
Source: PLoS One. 2013 Feb 22;8(2):e57416. doi: 10.1371/journal.pone.0057416 (PMC3579856; doi:10.1371/journal.pone.0057416)
Supplement: Table S1 — Short interfering RNA (siRNA) sequences. (DOCX) [file pone.0057416.s005.docx]

| **siRNA** | **Oligonucleotide sequences (5′→3′)** |
| --- | --- |
| B3GalT1 | Sense: CUGAUCCUGUUCUCAAUCAtt  Antisense: UGAUUGAGAACAGGAUCAGtt  Sense: CGAUGUAGCUGAACUCAUUtt  Antisense: AAUGAGUUCAGCUACAUCGtt  Sense: GUCAUAUCAUCCCUGCAAAtt  Antisense: UUUGCAGGGAUGAUAUGACtt |
| MUC1 | ACCUCCAGUUUAAUUCCU C |
