# Supplementary material for: Prostatic Cell-Specific Regulation of the Synthesis of MUC1-Associated Sialyl Lewis a
Source: PLoS One. 2013 Feb 22;8(2):e57416. doi: 10.1371/journal.pone.0057416 (PMC3579856; doi:10.1371/journal.pone.0057416)
Supplement: Table S2 — Oligonucleotide primers used for quantitative real-time PCR analysis. (DOCX) [file pone.0057416.s006.docx]

| **Target** | **Forward primer (5′-3′)** | **Reverse primer (5′-3′)** |
| --- | --- | --- |
| *C1GALT1* | AGAAGCAAAGGTCACCAGTCCCAA | AGCCAGGATTTAGAGGCCATTTCC |
| *GCNT1 (C2GnT-1)* | GCATGGTTTCTGCAGAGCACAGTT | TTTCCACAATGCCTTGCTACCTGC |
| *GCNT3 (C2GnT-2)* | AGAAGCGAGAGCCTTTCACAGACA | TACACAGCTCGCAGTAGCCTTTCA |
| *GCNT4 (C2GnT-3)* | GTTCCCTGGGCTTTCCTTGTTTGA | ACTCACCTAAGGCCAAAGGGAACA |
| *ST3Gal3* | TGTTCCTGGATGACTCCTTTCGCA | CTTGTTGGCAAGAACGCCTCCATT |
| *ST3Gal4* | ATGAGCAGATCACGCTCAAGTCCA | TCCCATCTCCAGCATCCGCTTAAT |
| *ST3Gal6* | TCTATTGGGTGGCACCTGTGGAAA | TGATGAAACCTCAGCAGAGAGGCA |
| *FUT3* | AGAGAGATCATCACGGCACGGTTT | ATAAGTGGTGGTCCTGGGCTTGAA |
| *FUT4* | TGCAGGTGGGACTTTGTTGTTTGG | TCCTCCAAGGACAATCCAGCACTT |
| *FUT5* | TGCCACACTGAATGTCACCTGCTA | TGCTGATGGTCACACACATACCCT |
| *FUT6* | ATCACCGCACCGTTTCCAGATGTA | TCCCAAAGTGCTGGGATTACAGGT |
| *FUT7* | TTCGTGCATGTGGATGACTTTGGC | AGCGTTGGTATCGGCTCTCATTCA |
| *FUT9* | TCCCATGCAGTTCTGATCCAT | GAAGGGTGGCCTAGCTTGCT |
| *B3GalT1* | GACCATGCTTGATTTCCTGAAC | CCTCTGCGCTCATTCTACTTTC |
| *B3GalT2* | AGTGGGCATAATCCTCTTCAC | GCAATCATTTTCTAATTCAGTCACATT |
| *B3GalT4* | CGCTATTCTTGCTGGGAGAG | GGGTCTTTAGGGTGAGGTTG |
| *B3GalT5* | ATTGGCATATCTGGTTCTGAGG | AGGTTCTGGGCTTGGTTTG |
| *MUC1* | TGTTACGGGTTCTGGTCATG | GTCGGTGCTGGGATCTTC |
| *MUC4* | GTCCTATGCCCTGTTTCTCTAC | CGATACCTCTCCCACACTG |
| *MUC16* | CTGAGACCCCAACATCCTTG | GGTCACTAGCGTTCCATCAG |
| *MUC17* | AGAACTGTAACCTCGGCAAG | CTGTACCAGTGAGTTTCCGTG |
| *GAPDH* | GAGAGGAGCAGGTAGAGGG | AGAATGAGGGTGGCATTGG |
| *GALNT1* | GGTATCCTGTTCCCCAAAGAG | CCCAAATATCCATTCCAGCATC |
| *GALNT2* | ACTAACTGCCTCGACACTTTG | GCTTCACCGACTTCTCCTTC |
| *GALNT3* | GACAGGATTTCTTTGCACCG | ACAGTTCTAAGCAACGTGGAC |
| *GALNT4* | ACCTTGGAACGTATGACACAG | TGCCACACCCTAAAAGACAG |
| *GALNT5* | ACCTTAGCGATTTGATCCCAG | CACACTTCATCCACAAAGCAC |
| *GALNT6* | GCACTGTCACAACTTTTCCTG | TTGTTCTCACCCACATCCAG |
| *GALNT7* | CGAAGTATTGGTGCTCAGAAGG | TGACATCTATAAGCGGCACAG |
| *GALNT8* | ACATCTGAATAAACGCTACGGG | ACTTCATCTTTCGCCCTCTTC |
| *GALNT9* | AAGTGTGAGGATGTGGCG | TGGCATCTTTGGACATCTCC |
| *GALNT10* | TTTTCTTGGGAGATGGGCAG | TTTTCTCGGTATGCCTGATCC |
| *GALNT11* | GCACTTCAAATGGGATCTTGTC | CCATGCCACTATCATACTGTCC |
| *GALNT12* | AAGTGGTTCTTGGAGACTGTG | AATCTGGTTTTCATCGGGAGG |
| *GALNT13* | GGTATCCTGTTCCCCAAAGAG | CCTGCATCGTAAGTTCCTATCTC |
| *GALNT14* | TTCGGGAATGTTGAGAGCAG | TTCTGTCTCTGTCGGATATTGC |
